# mTOR pathway in human cardiac hypertrophy caused by LEOPARD syndrome: a different role compared with animal models?

**DOI:** 10.1186/s13023-019-1204-4

**Published:** 2019-11-13

**Authors:** Hao Cui, Lei Song, Changsheng Zhu, Ce Zhang, Bing Tang, Shengwei Wang, Guixin Wu, Yubao Zou, Xiaohong Huang, Rutai Hui, Shuiyun Wang, Jizheng Wang

**Affiliations:** 1 Department of Cardiovascular Surgery, Fuwai Hospital, National Center for Cardiovascular Disease, Chinese Academy of Medical Sciences and Peking Union Medical College, 167 Beilishi Road, Xicheng District, Beijing, 100037 China; 2 Department of Cardiology, Fuwai Hospital, National Center for Cardiovascular Disease, Chinese Academy of Medical Sciences and Peking Union Medical College, Beijing, China; 3 State Key Laboratory of Cardiovascular Diseases, Fuwai Hospital, National Center for Cardiovascular Disease, Chinese Academy of Medical Sciences and Peking Union Medical College, 167 Beilishi Road, Xicheng District, Beijing, 100037 China

**Keywords:** LEOPARD syndrome, PTPN11, Hypertrophic cardiomyopathy, mTOR, Rapamycin

## Abstract

**Background:**

Animal studies suggested that blocking the activation of the mammalian target of rapamycin (mTOR) pathway might be effective to treat cardiac hypertrophy in LEOPARD syndrome (LS) caused by *PTPN11* mutations.

**Results:**

In the present study, mTOR pathway activity was examined in human myocardial samples from two patients with LS, four patients with hypertrophic cardiomyopathy (HCM), and four normal controls. The two patients with LS had p.Y279C and p.T468 M mutations of the *PTPN11* gene, respectively. Although *PTPN11* mutation showed initially positive regulation on phosphoinositide 3-kinase, overall the mTOR complex 1 pathway showed widely attenuated activity in LS. This included mildly hypophosphorylated mTOR and ribosomal protein S6 kinase and significantly hypophosphorylated Akt^308^ and ribosomal protein S6, which is similar to HCM. Akt^473^ is a basal molecule of the mTOR complex 2 pathway. Akt^473^ was less affected and showed hyperactivity in LS compared with HCM and normal controls. Additionally, MAPK/ERK kinase and ERK1/2 were significantly more phosphorylated in both HCM and LS than normal controls.

**Conclusions:**

In LS, the mTOR signaling pathway shows similar activity to HCM and is attenuated compared with normal controls. Thus, caution should be applied when using rapamycin to treat heart hypertrophy in LS.

## Background

**Fig. 1:**
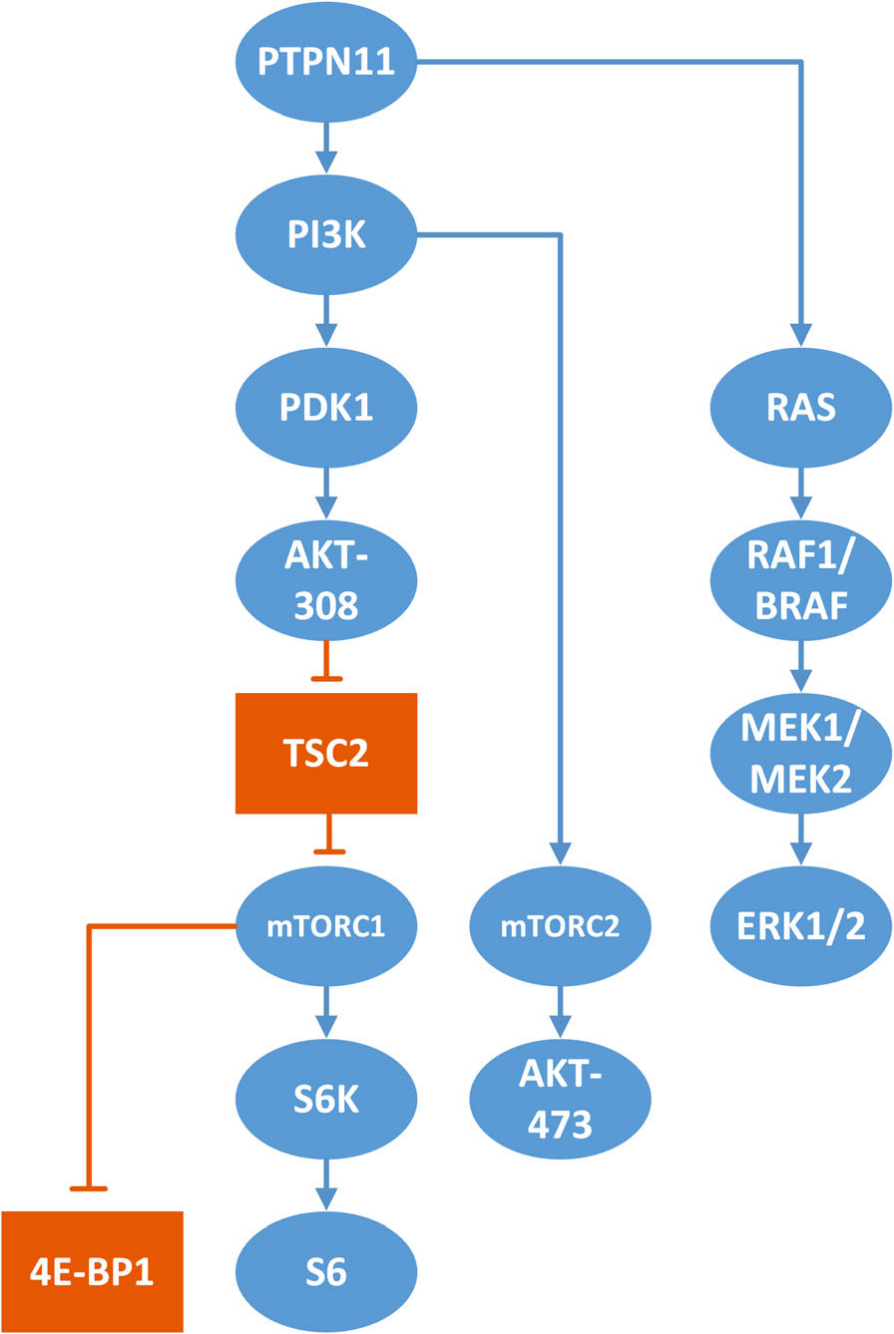
Signal pathways researched in the present study. A blue oval shape indicates the protein is a positive regulator in the pathway; an orange rectangle indicates the protein is a negative regulator. An arrow indicates an enhancing regulation on regulated protein; a blunt indication means an inhibitory regulation

LEOPARD Syndrome (LS) is a very rare autosomal dominant disease, and named for its major symptoms of *L*entigines, *E*lectrocardiography conduction abnormalities, *O*cular hypertelorism, *P*ulmonic stenosis, *A*bnormal genitalia, *R*etardation of growth, and sensorineural *D*eafness [1, 2]. Since it was first reported by Zeisler and Becker in 1936, only a few hundreds of patients have been reported worldwide. PTPN11 mutation is the most common cause of LS and accounts for approximately 90% of the patients with LS. PTPN11 gene encodes protein tyrosine phosphatase Shp2, which mediates several cellular signaling pathways (Fig. 1). Loss-of-function mutation of Shp2 protein in LS leads to the pleiotropic manifestations aforementioned. So far, at least 9 mutations involving 7 amino acid sites have been reported. These mutations may affect different functions of the Shp2 protein. More interestingly, gain-of-function mutations of PTPN11 also lead to a systemic presentation that overlaps quite much with syndrome caused by loss-of-function mutations. Therefore, understanding the abnormalities of signaling pathways will be vital in the treatment of LS.

Aside from the characteristic features, patients with LS are also commonly complicated by hypertrophic cardiomyopathy (HCM). HCM is an early-onset complication which may affect the survival of the patients with LS [3, 4], thus the prevention of cardiac hypertrophy is vital in the management of LS. In mouse LS model induced by *PTPN11* p.Y279C mutation, the activity of mammalian target of rapamycin (mTOR) activity was increased. Rapamycin inhibition of mTOR activity can reverse cardiac hypertrophy in *PTPN11*-mutated LS mice [5]. This result was also observed in animal studies on p.Q510E mutation. Thus, enhanced activity of mTOR was thought to be responsible for systemic manifestations, including HCM [6, 7].

In a later study, exploratory treatment using rapamycin analog improved heart function in an LS infant with severe cardiac hypertrophy [8]. The cardiac hypertrophy, however, was not reversed and resulted in heart transplant. Moreover, patients with gain-of-function mutations of PTPN11 and attenuated mTOR pathway may also have cardiac hypertrophy, which cannot be explained by the animal studies. This raised the concern that the abnormalities observed in animal models might not be the primary cause of cardiac hypertrophy in human. Evidence from human myocardial samples is warranted to understand the poor treatment effect of rapamycin.

## Results

**Fig. 2:**
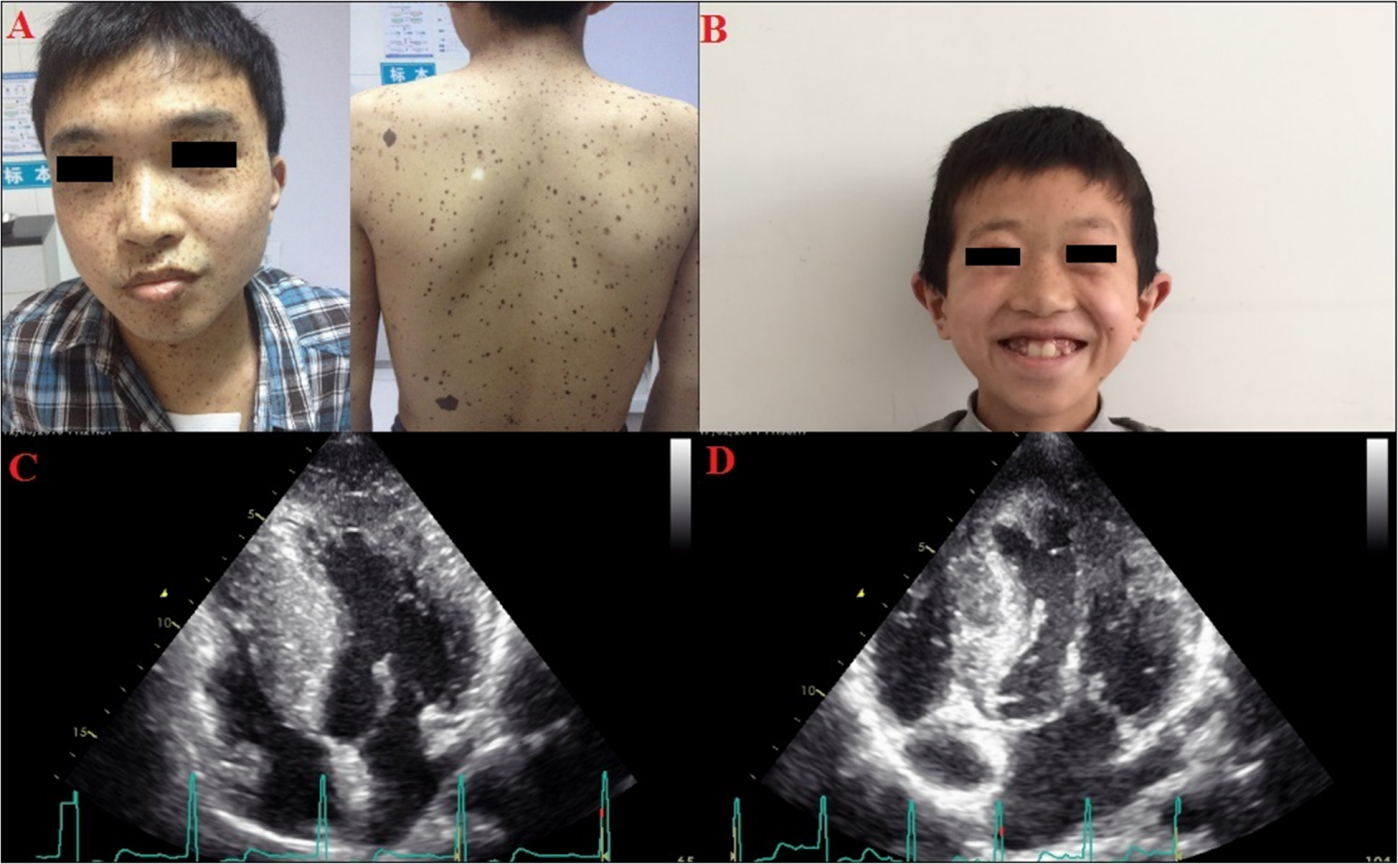
Facial features and echocardiography of two patients with LS. Panel (**a**)shows vast lentigines in patient LS1. Panel (**c**) shows the echocardiography of LS1, which reveals heart hypertrophy. Panel (**b**) shows the facial characteristics of patient LS2. Panel (**d**) shows the echocardiography of LS2, which also indicates heart hypertrophy

The first LS patient (LS1) was a 20-year-old male. He presented with multiple lentigines in the face, body, and limbs (Fig. 2a). Echocardiography showed biventricular hypertrophy and double outflow tract obstruction (Fig. 2c). Electrocardiogram showed ST-T segment changes and left ventricular hypervoltage without conduction block. The second LS patient (LS2) was a 10-year-old male. He had typical facial features of LS syndrome including multiple lentigines, hypertelorism, flat nasal bridge, and low-set ears (Fig. 2b). Echocardiography showed asymmetric left ventricular hypertrophy and left outflow tract obstruction (Fig. 2d).

Four male patients with HCM who underwent myectomy were included as positive controls. They were a similar age to the two LS patients: 34, 15, 20, and 13 years old. All the patients with HCM had no concomitant cardiac disease such as atrial fibrillation, coronary artery disease, or intrinsic valvular dysfunction. All patients with LS or HCM were using beta-blockers for symptom relief before septal myectomy.

The four patients with HCM were known to have myosin binding protein C, cardiac (*MYBPC3*) or myosin heavy chain 7 (*MYH7*) mutations before they were included (Table 1). Whole-exome sequencing found a heterozygous c.A836G (p.Y279C) *PTPN11* mutation in LS1 and a heterozygous c.C1403T (p.T468 M) *PTPN11* mutation in LS2. Subsequent Sanger sequencing validated both mutations. Further, both mutations had previously been shown as causative for LS by multiple studies [9]. No pathological mutations in other LS-related genes (namely, *RAF1* and B-Raf proto-oncogene, serine/threonine kinase [*BRAF*]) were found in the patients.

**Table 1:** Genetic background of LS patients and HCM controls

Patients	Age (years)	gender	gene	mutation	septal thickness(mm)
LS1	20	male	PTPN11	p.Y279C	33
LS2	10	male	PTPN11	p.T468 M	31
SA1	34	male	MYH7	p.A355T	26
SA2	15	male	MYH7	p.R403L	34
SA3	20	male	MYBPC3	p.D770N	43
SA4	13	male	MYBPC3	p.R495Q	41

*LS* LEOPARD syndrome; *SA* sarcomere-mutated hypertrophic cardiomyopathy

**Fig. 3:**
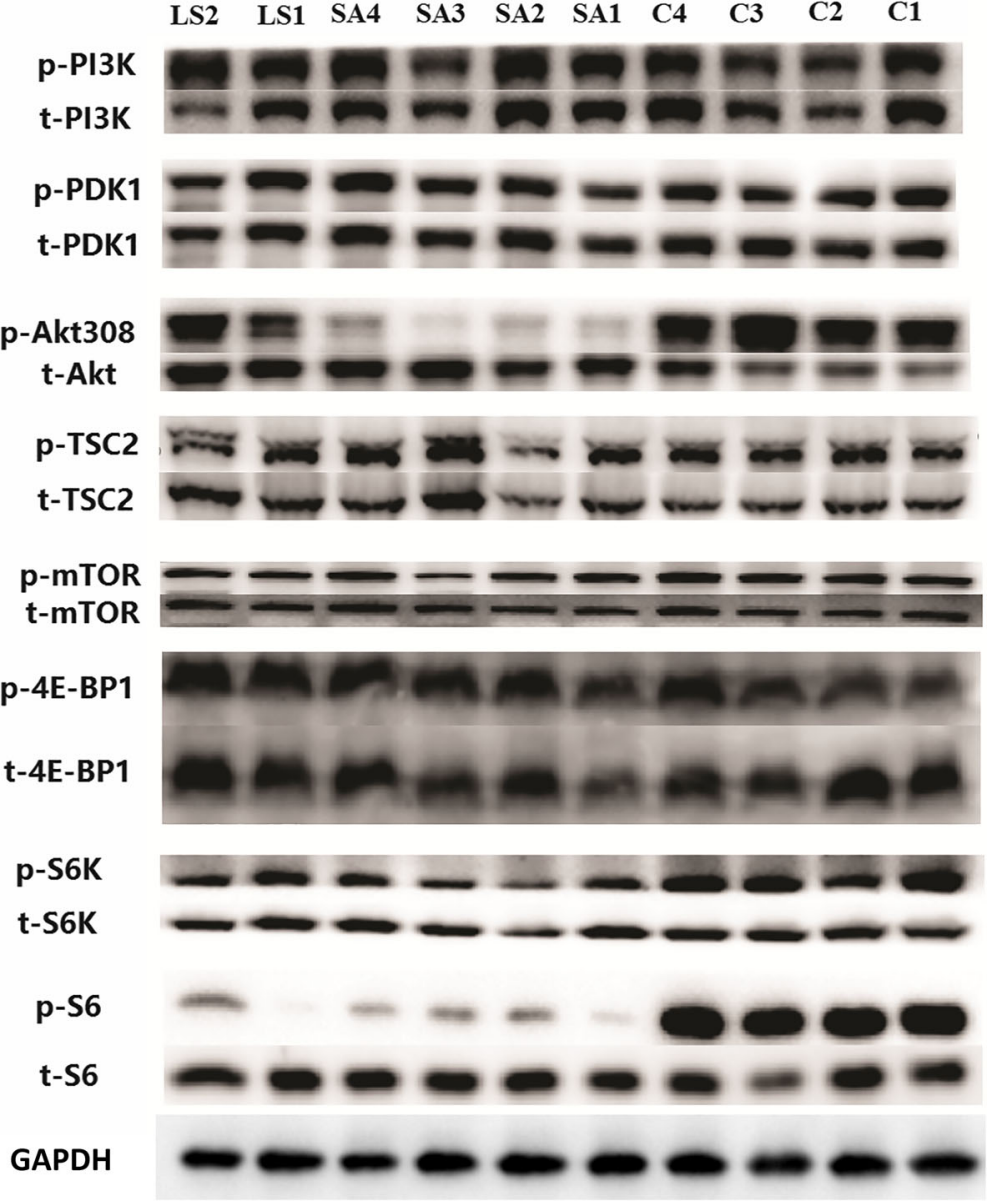
Signaling activity of the mTOR complex 1 pathway examined by western blotting. LS, LEOPARD syndrome; SA, sarcomere mutated HCM; C, healthy controls. P indicates phosphorylated protein, and t indicates total protein. PI3K, phosphatidylinositol-4,5-bisphosphate 3-kinase (gene name PIK3CA, PIK3CB, PIK3CG, and PIK3CD); PDK1, phosphoinositide-dependent kinase 1 (gene name PDK1); Akt, protein kinase B (gene name Akt1); TSC2, tuberous sclerosis complex 2 (gene name TSC2); mTOR, mammalian target of rapamycin (gene name MTOR); 4E-BP-1, eukaryotic translation initiation factor 4E-binding protein 1 (gene name EIF4EBP1); S6K, ribosomal protein S6 kinase (gene name S6k); S6, ribosomal protein S6 (gene name RPS6); GAPDH, glyceraldehyde 3-phosphate dehydrogenase (gene name GAPDH), was used as a loading control

**Fig. 4:**
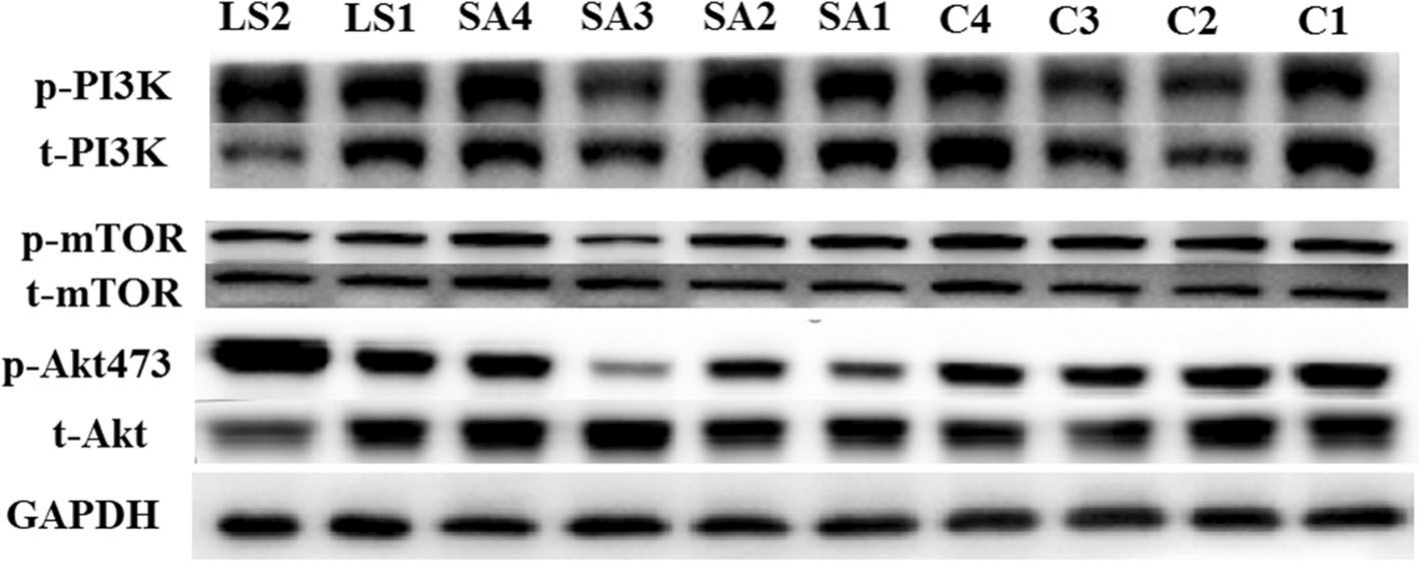
Signaling activity of the mTOR complex 2 pathway. LS, LEOPARD syndrome; SA, sarcomere mutated HCM; C, healthy controls. P indicates phosphorylated protein, and t indicates total protein. PI3K, phosphatidylinositol-4,5-bisphosphate 3-kinase (gene name PIK3CA, PIK3CB, PIK3CG, and PIK3CD); mTOR, mammalian target of rapamycin (gene name MTOR); Akt, protein kinase B (gene name Akt1); GAPDH, glyceraldehyde 3-phosphate dehydrogenase (gene name GAPDH), was used as a loading control

**Fig. 5:**
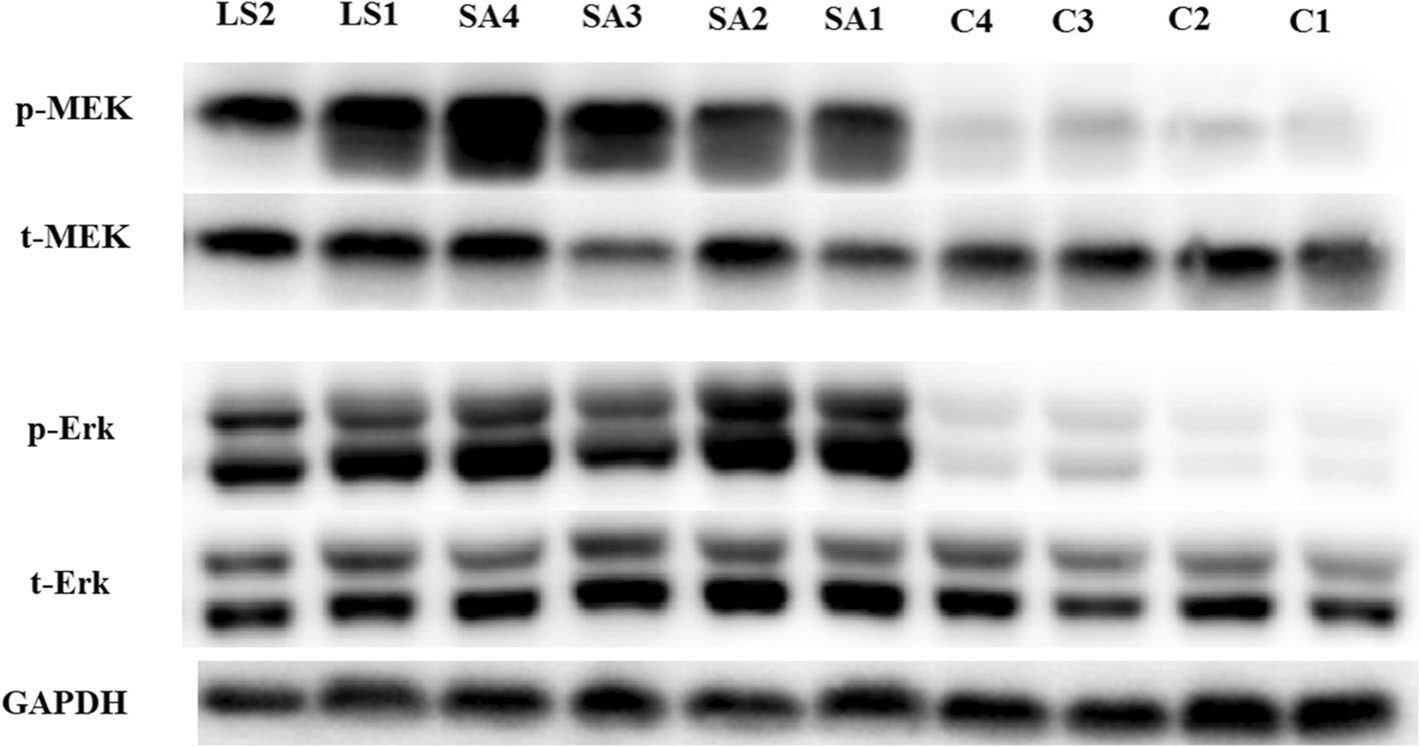
Signaling activity of Erk1/2 pathway. LS, LEOPARD syndrome; SA, sarcomere mutated HCM; C, healthy controls. P indicates phosphorylated protein, and t indicates total protein. MEK, mitogen-activated protein kinase (MAPK)/extracellular signal-regulated kinase (ERK) kinase (gene name MAP2K1 and MAP2K2); ERK, extracellular signal-regulated kinase (gene name MAPK1); GAPDH, glyceraldehyde 3-phosphate dehydrogenase (gene name GAPDH), was used as a loading control

As shown in Fig. 3, PI3K was hyperphosphorylated in LS but its downstream effector PDK1 was approximately the same. Akt^308^ was markedly hypophosphorylated in the HCM group, yet only slightly hypophosphorylated in patients with LS compared with normal controls. This led to subsequent mild hypophosphorylation of mTOR in LS and HCM. Consequently, targets of the mTOR complex (mTORC)-1, S6K and 4E-BP1, were slightly changed. However, S6 was significantly hypophosphorylated in both the LS and HCM groups. Although initially enhanced, the mTORC1 pathway gradually became attenuated during subsequent signal transduction. Akt^473^, a target site of mTORC2, was hypophosphorylated in the HCM group and slightly hyperactivated in the LS group (Fig. 4). Our results also showed hyperphosphorylated ERK1/2 in the HCM and LS groups (Fig. 5). Moreover, their regulatory molecules, MEK1/2, were also significantly hyperphosphorylated.

## Discussion

The present study is the first and the only study on the human sample with PTPN11-mutated Leopard syndrome. Our findings were different from the results from animal models [5]. mTOR/Akt/S6K pathway was not enhanced in human myocardial samples from LS patients, in contrast to the animal models. Moreover, MEK/Erk was significantly enhanced in the present study but showed attenuation after stimulation in the animal model.

LS is also called Noonan Syndrome with Multiple Lentigines because it overlaps with Noonan Syndrome in multiple phenotypes. Indeed, it also shares the main causative gene (*PTPN11*) with Noonan Syndrome, although the underlying molecular mechanisms are entirely different. *PTPN11* mutations in LS are loss-of-function mutations, while those in Noonan Syndrome are gain-of-function mutations [9–11]. Interestingly, both types of *PTPN11* mutations can lead to HCM.

The two patients with LS in our study manifested typical features of LS. T468 M and Y279C of *PTPN11* are the most common mutations in LS [12]. Previous studies have shown that enhanced activity of mTORC1 is a key factor of HCM in LS. Rapamycin can reverse HCM in a mouse model of LS. Moreover, although a later attempt of rapamycin treatment in an infant patient did not change the final outcome, the symptoms were greatly alleviated. Thus, the efficacy of rapamycin treatment in humans remains to be validated. In previous studies, hyperphosphorylated Akt^473^ and S6K^389^ were regarded as evidence of elevated mTORC1 activity [5, 6]. According to some studies, however, Akt^473^ is a substrate of mTORC2, and Akt^308^ is the true catalytic site of the mTORC1 pathway [13]. Our study showed that Akt^308^ was hypophosphorylated in hypertrophic myocardial tissue, which might change the initial hyperactive effect of *PTPN11* mutation. Downstream signaling molecules, including mTOR and S6K, were slightly hypoactivated due to attenuated Akt^308^ activity. Additionally, our data showed marked hypophosphorylation of S6 in both sarcomere-HCM and LS. This finding implicates multiple branching downstream factors in the regulation of PTPN11/PI3K/Akt/mTORC1. Activated S6K also has a negative feedback effect on insulin receptor substrate 1, which is an upstream regulator of PI3K. Altogether, hypertrophic pathophysiology may have a greater impact on this signaling pathway than mutation.

Rapamycin was reported to be associated with suppressing various types of cardiac hypertrophy, regardless of primary hyperactivity of mTORC1 [14, 15]. In contrast, *RAF1* and *BRAF* mutations can also lead to LS [1, 16, 17]. These two proteins are components of the RAS/RAF/ERK pathway, which is also regulated by PTPN11. Theoretically, they do not have a significant impact on mTORC1 activity. These findings suggest that *PTPN11* mutation may induce LS beyond the mTORC1 pathway, while primary hyperactivation of mTORC1 is not a prerequisite for the hypertrophy-reversing effect of rapamycin. In this observational study, differences between LS1 and LS2 also showed that the functional status of different *PTPN11* mutations might be distinct, which raises the concern that determining the dose of rapamycin would be hard in clinical practice.

Basal substrates of the RAS/RAF pathway were also examined. Unexpectedly, MEK and ERK were significantly hyperphosphorylated in both LS and sarcomere-HCM, which is another significant paradox. LS-related *PTPN11* mutations reportedly diminish ERK activation, while in our study, hypertrophic myocardial samples presented enhanced ERK activation [12, 18]. The lowest molecules of this pathway showed similar changes in LS and sarcomere-HCM. Consequently, branching regulations are strong enough to reverse the primary catalytic alteration. Considering the same changes in a different genetic background, the role of the RAS/RAF pathway in hypertrophy cannot be ignored. A previous study reported that calcium signaling dysregulation might play a role in both sarcomere and non-sarcomere HCM. These intricate signaling pathways are widely dysregulated in myocardial hypertrophy and can further affect each other. As a result, effector molecules might not just behave as theories.

Although gene mutation is the primary cause of the LS, straight-forward analysis of the downstream signaling may be misleading. PTPN11 gene and downstream pathways widely involve many metabolic activities and are also regulated by these metabolic molecules. The homeostasis of the signaling network is a balance between gene function and environmental stimulation. Functional studies on model animals cannot reflect the real pathogenic mechanism in human beings. Using metabolomic methodology to unveil what is happening in the cells may help our understanding of the pathophysiology in LS [19].

This is an observational study on human heart samples. Lack of a functional study attenuated its power. Meanwhile, only two LS patients were included in the study, which limited statistical analysis of changes. Cardioplegia might have a potential effect on the studied pathways. Further, due to limited samples, the pathways involved were not complete for *PTPN11* mutations, and valuable information remains to be revealed. Study results from this observational study cannot completely refute the hypothesis set up by a previous study, although our findings show a contradiction in pathway presentation. More convincing evidence from human samples is needed to fully elucidate the mechanism of LS.

## Conclusions

In patients with LS, the mTOR signaling pathway shows similar activity to HCM and is attenuated compared with normal controls. Rapamycin treatment for LS should be cautious, especially for patients with LS who have developed cardiac hypertrophy.

## Materials and methods

Two patients were presumptively diagnosed with LS based on their clinical manifestation and family history. Subsequent gene sequencing confirmed the diagnosis. They underwent septal myectomy because of left ventricular outflow tract obstruction. Myocardial samples from four gender- and age-matched obstructive HCM patients with pathogenic sarcomere mutation were included as HCM controls. Myocardial specimens were resected during surgical operation, and immediately cut and preserved in liquid nitrogen. Myocardial samples from four healthy donors who had accidental deaths were included as healthy controls. Left ventricular muscle was used for protein analysis.

DNA was isolated from myocardial tissues of the presumptive patients with LS, and whole-exome sequencing was performed to identify causing mutation of the disease. Pathogenic mutations were subsequently validated by Sanger sequencing. The sarcomere mutations of HCM controls were detected by panel sequencing as described previously [20] and confirmed by Sanger sequencing.

Myocardial samples were lysed on ice in lysis buffer (CWBiotech, Beijing, China) containing protease inhibitor (Roche, Basel, Switzerland) and protein phosphatase inhibitor (Roche). Extracted proteins were preserved at − 80 °C for use after quantification and denaturation. Prepared protein samples were separated by sodium dodecyl sulfate-polyacrylamide gel electrophoresis. Samples were then blotted onto polyvinylidene fluoride membranes with a bore diameter of 0.22 μm (Merck Millipore, Darmstadt, Germany). Membranes were blocked with 5% nonfat milk in Tris-buffered-saline with Tween-20 at room temperature. Next, the membranes were incubated overnight at 4 °C with primary antibodies. Subsequently, the membranes were incubated with horseradish peroxidase-conjugated secondary antibodies for 1 h at room temperature. Specific bands were detected using the SuperSignal West Femto Maximum Sensitivity Substrate (Pierce, Rockford, IL, USA). Glyceraldehyde 3-phosphate dehydrogenase was used as the reference protein. The following protein and phosphorylation sites were examined with the corresponding primary antibodies: total phosphatidylinositol-4,5-bisphosphate 3-kinase (PI3K) p85, phospho-PI3K p85^458^, total phosphoinositide-dependent kinase 1 (PDK), phospho-PDK^241^, total Akt, phospho-Akt^308^, phospho-Akt^473^, total tuberous sclerosis complex 2 (TSC2), phospho-TSC2^1462^, total mTOR, phospho-mTOR^2448^, total ribosomal protein S6 kinase (S6K), phospho-S6K^389^, total ribosomal protein S6 (S6), phospho-S6^235/236^, total eukaryotic translation initiation factor 4E-binding protein 1 (4E-BP1), phospho-4E-BP1^37/46^, total mitogen-activated protein kinase (MAPK)/extracellular signal-regulated kinase (ERK) kinase (MEK1/2), phospho-MEK1/2^217/221^, total ERK1/2, phospho-ERK1/2^202/204^, and glyceraldehyde 3-phosphate dehydrogenase. All antibodies were from Cell Signaling Technology, Danvers, MA, USA. The pathway examined is shown in Fig. 1 [13, 21].

## Data Availability

The datasets used and/or analysed during the current study are available from the corresponding author on reasonable request.

## Abbreviations

HCM: Hypertrophic cardiomyopathy
LS: LEOPARD syndrome
